# Adhesion of Modified Epoxy Resin to a Concrete Surface

**DOI:** 10.3390/ma15248961

**Published:** 2022-12-15

**Authors:** Andrzej Szewczak, Grzegorz Łagód

**Affiliations:** 1 Faculty of Civil Engineering and Architecture, Lublin University of Technology, Nadbystrzycka 40, 20-618 Lublin, Poland; 2 Faculty of Environmental Engineering, Lublin University of Technology, Nadbystrzycka 40B, 20-618 Lublin, Poland

**Keywords:** adhesion, epoxy resins, profilometry, sonication, microsillica, carbon nanotubes, durability, surface protection, building material

## Abstract

The protection of building elements exposed to the weather using hydrocarbon-based agents is a comprehensive group of analyses. These agents are characterized by very high chemical resistance, waterproofness, as well as adhesion to surfaces made of various materials, i.e., concrete, steel, ceramics and wood. Modification of adhesion, which ultimately leads to an increase in the durability of a protective/face coating made of such a material, can lead to a longer life of these layers and a less frequent need for replacement or restoration. The following paper describes an experimental research program on the possibility of increasing the adhesion and durability of epoxy resin modified with the use of powder fillers. The resin can be used as a protective or top coat on the surface of concretes or mortars. The main objective of the study was to increase the adhesion of the resin to the concrete substrate, modified by grinding and sandblasting to increase the roughness. For the series studied, both the changes in physicochemical parameters, which determine how the resin penetrates the irregularities of the substrate and mechanical parameters, which mainly determine the durability of the layer made in this way, were identified. A modified version of the pull-off test was used as a method to directly evaluate the effectiveness of the modified resins.

## 1. Introduction

Polymers are a group of compounds that find a variety of applications in numerous areas of everyday life. Their main feature is their diverse physical, physicochemical, rheological, processing or electrical properties, which allow their use in many sectors of industry, medicine and construction [1,2,3]. Among the numerous types of polymers in structural engineering, the most commonly mentioned are [4,5,6,7,8,9,10,11,12,13,14]:

- *Fiber-forming polymers*—a group of polymers with reinforcement in the form of fibers (glass, carbon, aramid, basalt, etc.);
- *Structural polymers (resin concretes)*—used as a construction material in which the cement binder is partially or completely replaced by a polymer;
- *Coating polymers:*
  (a) Hydrophobic (impregnating) polymers in the form of agents applied as coatings are used to protect porous materials;
  (b) Paints and varnishes—a group of polymeric compounds used as paint coatings, as a decorative element, for interiors and facades of buildings;
  (c) Protective coatings, films—films have become very popular in the construction industry, as materials used to protect other elements, waterproofing, shielding, protective coatings;
- *Insulating materials*—this group includes Styrofoam, one of the most popular materials currently used for thermal insulation and thermal upgrading of buildings;
- ***Adhesive polymers (glues)***—epoxy, polyester, phenolic, formaldehyde, polyurethane, resins [15,16,17,18,19]; this is a group of polymers which, owing to their very good adhesive properties and high adhesion to various materials, i.e., steel, concrete, wood, ceramics, have found their use for joining or reinforcing elements; adhesives can be used for secondary bonding of reconstructed or detached defects of elements, bonding of layers, also made of different materials; an important feature of adhesives is their quick setting, very good adhesion, low shrinkage and creep and minimal tendency to relax during operation of the bonded joint.

In the case of external building partitions and elements, there is a legitimate need for polymer-based adhesives for bonding and joining elements, such as facade panels. It is also possible to manufacture protective and usable surface coatings for terraces and balconies. Facades, terraces and balconies are particularly exposed to atmospheric conditions, among which changes in humidity and temperature have the most adverse effect on the durability of these elements. Variable humidity promotes biological corrosion, which is often largely responsible for the degradation of outdoor building elements, especially on the northern facade, which is most exposed to adverse weather conditions. In addition, the moisture that penetrates into the deeper layers of a partition, such as a wall or terrace, can, under the influence of low temperature, accelerate the deterioration of such elements, especially when they are below the freezing point. The issue of degradation of external parts of buildings caused by weather conditions is the subject of numerous publications [20,21,22,23,24,25]. It is also important to provide protection against possible chemical compounds formed when water vapor comes into contact with compounds, such as sulfur, nitrogen and phosphorus contained in the air. These can affect the slow degradation of cladding panels and top coatings of flat surfaces due to the occurrence of chemical corrosion [21,26]. The occurrence of damage to the outer layers of the building envelope leads to the need for repair, replacement or restoration. Each of these operations generates additional costs associated with the operation of buildings that can be avoided.

A common way to protect facades, terrace surfaces or balconies against adverse weather conditions is to use cladding panels, made of stone, ceramic, wood or plastic [27,28]. In the case of facades and plinths, it is possible both to stick such elements with adhesives and with the use of various mechanical fasteners, i.e., anchors, sleeves, frames, brackets, hangers. Facade cladding panel elements, in addition to their protective function, frequently constitute an element that affects the building aesthetics, often having an additional decorative and artistic function [27]. On the other hand, on flat surfaces, cladding—which in addition to protecting the deeper layers, also provides the possibility of safe movement on them—is attached with adhesives. The classic examples of such materials are ceramic and stone tiles [28]. It is also possible to make the floor of the terrace and balcony a homogeneous layer. Unfortunately, the large climate variation associated with the occurrence of seasons with substantial fluctuations in humidity and temperature can adversely affect both the condition of the cladding itself and the adhesive layer. Traditional adhesives based on mortars containing cement and other water-based binders, despite fulfilling their function, are at greater risk of water penetration from water vapor contained in the air or penetrating under the cladding panels as a result of the occurrence of, for example, leaks in the joints between the elements. Gradual degradation of the adhesive layer can also be caused by corrosion. As a result, after some time, the cladding layer may become detached from the substrate along with the adhesive layer [22,29,30].

**Figure 1:**
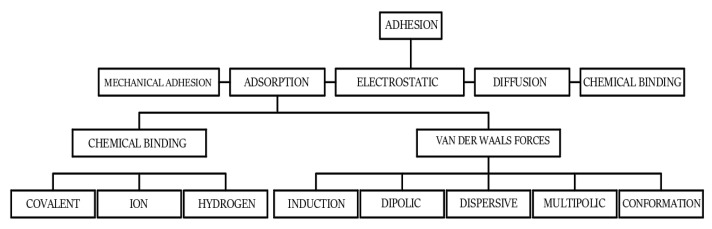
Adhesion models.

Considering all of these aspects, it seems justified to look for methods to avoid them. One such method is the use of polymeric adhesives as materials for gluing elements of facades and plinths, terraces and balconies. Their main characteristics are chemical resistance, significant adhesion to various types of substrates and non-absorbability [1,2,3]. Nevertheless, these materials also have their drawbacks. The most important one is the degradation of the polymer layer caused by the so-called aging process, associated with the gradual weakening of the chemical bonds between the molecules that make up the polymer [1,2,5,14]. This process can be accelerated by the effects of UV radiation, causing the release of free radicals from the polymer structure. In this situation, methods of modifying polymers are being sought to increase their durability [2,3,31,32]. The first group of analyses considers the possibility of improving the wettability of a substrate, such as concrete, by the polymer and its easier penetration into its rough surface during adhesive application. This is possible mainly due to the changes in wetting angle and surface free energy, which is of particular importance in the case of polymers [33,34,35]. This quantity, which is composed of a dispersive and polar component, accurately describes the bonding of the adhesive to the substrate from the point of view of the occurrence of physicochemical phenomena [35]. Most often, it is also possible to increase the peel strength from the substrate and change the modulus of elasticity. These two parameters largely determine the stiffness and, consequently, the durability of the adhesive between, for example, a stone slab and the surface to which the slab is adhering [36]. Adhesion itself is a complex phenomenon that does not have a homogeneous definition. The models shown in Figure 1 are the types of adhesion accepted and described in the literature [15,18].

When gluing cladding panels to different types of substrates, the main types of adhesion that will determine the effectiveness of such a joint are mechanical and adsorptive adhesion. Changes in the adhesion of a polymer are made possible by modifications to its structure. One such modification is the use of fillers in powder form. This is a group of agents that, when intentionally added to a polymer, have a beneficial effect on improving its parameters, mainly mechanical, processing or adsorption. Special additives affecting the electrical and thermal conductivity of various types of polymers are also described [5,35,37,38]. From the point of view of improving the durability of bonded joints, the fillers belonging to the first group are the most important. The fillers that are most commonly described in the literature and studied and that modify polymers in terms of achieving changes in mechanical parameters include silica, powdered metals and their oxides, graphene, carbon nanotubes, carbon black, zeolites, microsilica, cellulose (which has microfibers despite its relatively powdery structure), dolomite, limestone and chalk. The amount of filler is most often determined by the amount of adhesive to which the filler is added. Particle size is also of great importance. Variation in the results of the effectiveness of a given modification is possible when using the same filler, but in the form of micro- or nanoparticles [39]. For this reason, it is important to carefully examine the effect of a given filler on the specific, altered initial properties of the adhesive. In addition, the method of distribution of filler particles in the adhesive (resin) volume is very important. For this purpose, mechanical mixing, ultrasound, pressure and heating methods described in the literature are used. Filler additives can favorably affect the adhesion of the adhesive to a given substrate and in the case of polymers also affect their resistance to aging caused by the slow decomposition of the chemical structure. Among other modification methods described in the literature, there are those involving the treatment of the polymer with elevated temperatures induced by radiation from a specific source, i.e., microwave, electron, infrared radiation in the presence of additional catalysts and chemical reaction inhibitors, such as organic peroxides or inorganic compounds [40,41].

The corresponding effect is also achieved by varying the roughness of the substrate, which leads to a system in which adjacent layers (adhesive and substrate) can interlock and wedge with each other according to the assumptions of the mechanical adhesion model. The definition and model of mechanical adhesion were first described by McBain and Hopkins [42]. Roughness should be understood as the variation of the state of roughness of the substrate, which is described in the literature by means of relevant parameters [43,44]: the maximum elevation of the roughness profile R_p_, the maximum depth of indentations of the roughness profile R_v_, the maximum height of the roughness profile (R_z_), the average height of the elements of the roughness profile R_c_, the total height of the roughness profile R_t_, the arithmetic mean deviation of the ordinates of the roughness profile from the mean line R_a_, the mean square deviation of the ordinates of the roughness profile R_q_, the coefficient of asymmetry of the roughness profile also called the skewness coefficient of the roughness profile parameter R_sk_, the coefficient of inclination of the roughness profile R_ku_ and the average width of the grooves of the roughness profile R_sm_.

Proper surface preparation can affect significant changes in the aforementioned parameters and the final increase in adhesion. In the case of surfaces to which it is possible to glue facade cladding, terraces and balconies, the most commonly used methods of their treatment are: surface cleaning to remove weak layers, bulk abrasive, abrasive-blasting, shot peening, brushing, grinding and milling [45].

The surfaces undergoing modification can then be tested to determine the aforementioned parameters using the following methods [46,47,48,49]: contact profilometry, optical profilometry, spectroscopy, such as scanning electron microscopy (SEM), Auger electron microscopy, slow electron diffraction (LEED), and tomography.

Adhesion by adsorption is also important in a bonded joint, which, according to Figure 1 depends mainly on the forces and chemical bonds between the atoms that make up the different phases [15,31,35]. In the case of surfaces made of various materials, e.g., concrete, ceramics, stone, the described surface treatments, in addition to the possibility of increasing roughness, also contribute to changing the energy state of the surface layer [15,45]. Changing the position of charges on the surface can lead to polarization and the formation of active centers, which more easily participate in chemical reactions with adhesive layers. The assumptions of the Lewis theory of acids and bases are important in the description of this phenomenon [50]. According to this theory, the substrate constitutes the base as the phase that is the electron donor, while the adhesive is the electron acceptor. The degree and amount of electrons thus exchanged and the bonds formed depend on the overall condition of the adjacent surfaces. However, in addition to proper surface preparation, the condition of the adhesive, which is applied to the substrate before bonding, for example, a ceramic plate, is also very important. Polymers, which include epoxy resins, are characterized by the presence of numerous functional groups in their structure. In addition, between the polymer chains there are the so-called free electron clouds, which, through appropriate modification of the polymers, can additionally participate in the exchange between the adhesive and the substrate. This effect is possible, for example, due to the use of ultrasound during adhesive preparation, as was also described in [10,11,35]. The ultrasonic cavitation, acting during sonication, leads to a gradual, temporary disruption of the originally arranged polymer chains, their relocation, changing the position of functional groups. The study shows that after stopping the sonication process, the adhesive returns to equilibrium in a relatively short time; however, it manifests greater ordering of its internal structure and chemical reactivity. This effect is associated with the release of electrons from the clouds to the outside and their further participation in the formation of chemical bonds, permanent and temporary, resulting from the presence of van der Waals forces. The processes occurring at the interface of phases subjected to bonding can be described in great detail owing to the scientific field of surface physicochemistry [15,18]. In addition to basic definitions, developed back in the first half of the 20th century, this field deals with very complex processes, many of which are precisely related to the use of polymers and their processing. The description of the phenomena occurring at the phase boundary is difficult due to its complex nature, which is also confirmed by the adhesion phenomenon. Nevertheless, the development of research in this field over the years has allowed a very good understanding of the essence of the behavior of molecules and atoms in the contact layer between two surfaces, which can be described by various quantities, such as surface tension, surface free energy, contact angle and viscosity. Numerous methods and approaches have been developed to study these phenomena. The most recent development, described in [51,52], among others, is the possibility of analyzing the connections between materials using molecular dynamics. Accurate analyses of molecular motions are performed, both predictable and probable, taking into account, for example, the complex state of stress in an adhesive joint subjected not only to mechanical loads but also to environmental factors. Three-dimensional simulation of the behavior of chemical molecules that build polymer compounds is possible by using the equations of state of motion of atoms and combining them in a complex way with equations of motion, such as all Newton’s equations of motion. To sum up, the factors affecting the effectiveness of bonding are [15,16,17,18,36,45]:

- Method of surface preparation and its parameters, roughness;
- The type of materials to be joined and the structure of their surfaces;
- The type of adhesive used, its method of preparation, modifications carried out in the liquid state and curing;
- The conditions under which the glued joint will operate;
- Mechanical properties of the adhesive and the substrate.

In the presented study, the main objective of the conducted analyses was to determine the effectiveness of the adhesive process with an adhesive modified with ultrasound and the addition of microsilica and carbon nanotubes on three types of concrete surfaces—cleaned, ground and sandblasted. Epoxy adhesive with the addition of quartz flour was selected for the study, which can be used both as an adhesive for bonding cladding panel elements to different types of substrates, as a putty for filling defects and making repairs, as well as for making homogeneous layers in the form of floors and protective coatings. The peel strength of CFRP (Carbon Fiber Reinforced Polymer) tape fragments glued to concrete was an indicator of the effectiveness of the applied modifications of the modified adhesive; it was tested using the pull-off method. The tests carried out enabled one to determine the changes occurring in the structure of the epoxy resin due to the application of microsilica and carbon nanotubes. Selected parameters of the adhesive in the liquid state, before curing, i.e., viscosity, wetting angle, temperature, were also used to determine the effectiveness of bonding. The scientific novelty of the work is a comprehensive analysis of the factors affecting the improvement of adhesion and, consequently, the durability of the layer made of the tested resin. It is important to use microsilica and carbon nanotubes for this purpose as additives which improve adhesion of the adhesive to the concrete surface. Average values and the coefficient of variation which is the ratio of the standard deviation to the mean values were defined as statistical parameters.

## 2. Materials and Methods

### 2.1. Components Used and Mixtures

The adhesive tested was the EP430 epoxy resin (Ciech Sarzyna, Nowa Sarzyna, Poland) with quartz flour as a base filler. Its main purpose is to glue elements made of various materials, such as cladding panels to flat or vertical surfaces. Using the adhesive, it is possible to make chemical- and frost-resistant floors for terraces, balconies or industrial plants. The resin compound can also be used as a putty or to fill in defects and damaged sections of walls and floors. It is also used to make floors in the form of a uniform layer laid on a base of concrete or cement or gypsum mortar. The important condition is to maintain a maximum moisture content of the substrate equal to 4%. The basic properties of the adhesive used in the study are shown in Table 1. In the adopted test program, the adhesive was used to adhere tiles cut from CFRP tape to the concrete surface. The purpose of the tests was to determine the adhesion of the adhesive to the concrete substrate, prepared according to three schemes: cleaned, ground and sand-blasted substrate. In addition, prior to the pull-off test, a series of tests were performed on the adhesive in a liquid state, before adding the hardener. A total of four series of samples, each consisting of eight readings from the pull-off device, were subjected to the pull-off test on the concrete surface. The choice of tiles made of such a material was based on the assumption of detaching from the concrete a material with as little thickness as possible. In this way, the thickness of the sample, as well as its weight at the time of sticking, did not significantly affect the adhesive positioning between the sample and the concrete substrate. It was also easier to regulate the flow of adhesive out from under the specimen at the time of bonding and curing.

The adhesive was prepared for testing according to the following scheme: unmodified adhesive, modification by ultrasound, modification by ultrasound with the addition of microsilica at 0.5% by weight of the resin and modification by ultrasound and with the addition of carbon nanotubes at 0.1% by weight of the resin. All tested formulas are shown in Table 2. For the latter two batches, ultrasound was used as a factor to enable both modification and mixing of the fillers into the adhesive. The fillers used were:

- BASF microsilica (BASF, Ludwigshafen, Germany), with a density of 2.2 g/cm^3^, mean particle size of 0.1 µm and a specific surface area of 20,000 m^2^/kg;
- NanocylTM NC7000 carbon nanotubes (NANOCYL, Sambreville, Belgium) with a density of 1.3–1.4 g/cm^3^, an average diameter of about 9.5 nm, a length of 1.5 μm and a specific surface area of 250–300 m^2^/g.

The main purpose of the filler additives was to improve the adhesion of the adhesives to the concrete substrate. In order to determine all possible changes caused by the addition of fillers, the parameters of the adhesive were analyzed both at the time of its application to the substrate (viscosity) and after hardening (mechanical parameters, adhesion).

The hardener used for crosslinking the adhesive was the Z1 amine (Ciech Sarzyna, Nowa Sarzyna, Poland) (triethyltetramine), the density of which at 22 °C is 0.98 g/cm^3^ while its viscosity is in the range of 20–30 mPa∙s. The amount of hardener was related to the total weight of the adhesive in the sample, according to the manufacturer’s recommendations.

Before the adhesive was subjected to modifications, its main initial parameters, i.e., viscosity at 22 °C, were determined. Prior to modifications, the density and viscosity of the unmodified resin were determined. The sonication time for each series was the same at 7 min. For the EP430/US series, this was the time of the modification carried out, while for the EP430/US/MS and EP430/US/NT series, it was the time of both modification and mixing the adhesive with the corresponding filler. The length of sonication was based on the preliminary tests conducted for the planned program. During its determination, the density of the adhesive, the addition of quartz meal in its initial formulation and the need to evenly distribute the particles of the fillers used in the set volume were taken into account. The observations made during sonication, mainly related to the highly dynamic phenomena resulting from rapid mixing of the mass of adhesive and fillers caused by ultrasonic cavitation, were also important. Sonication was carried out using the UP 400S desktop sonicator (Hielscher Ultrasonics Gmbh, Teltow, Germany), which has the ability to emit ultrasonic waves at a frequency of 24 kHz and an adjustable power range from 0 to 400 W. It is also possible to control the cycle (amplitude) in the range of 0.5–1 (50–100% of the amplitude value). The sonicator was turned off after 7 min, while the adhesive batch in question was subjected to further analysis. Since sonication causes an increase in the temperature of the adhesive, and thus a decrease in its initial viscosity, the changes in viscosity and temperature were measured at equal time intervals of 5 min. The end point of the measurements was when the adhesive reached the ambient temperature at which the initial parameters (density, viscosity) were measured, taken as 22 °C. A similar test program, for an adhesive intended for other applications and with other sonication parameters, was also described in [35,53].

To determine the adhesion of adhesives to concrete, the C30/37 class concrete was designed. Concrete is a material used for the construction of walls of buildings in monolithic technology, underground parts of buildings, as well as a building block for the construction of balconies and as a substructure (base) for layers of terraces and other flat surfaces associated with buildings. Its specific internal structure resulting from the fact that the essence of concrete as a composite containing coarse aggregate, fine aggregate, cement and water enables one to obtain a surface of varying roughness. The surface of concrete itself can also be easily modified, e.g., via abrasive methods. Since ordinary concrete is a porous material that absorbs water, it is important to protect it through the use of hydrophobizing agents or linings, as well as protective coatings made from compounds based on organic and inorganic polymers [23,28,54,55]. The concrete designed for testing was not subjected to additional analyses. The tests carried out after 28 days of maturation under water bath conditions on five 15 × 15 × 15 cm cubes, in accordance with the requirements of the standard [56], confirmed the assumptions made during the design of the concrete composition regarding its class. A mixture of granite and sand (sand point was assumed at 30%), as well as Portland cement CEM I 42.5R was used for the concrete. The composition of the concrete in relation to 1 m^3^ is as follows: granite—1438.2 kg; sand—389.9 kg; cement—393.3 kg; water—175.5 L.

Cubic specimens, which were not used in the study, served as a substrate onto which CFRP tape sections measuring 2.5 × 3.0 cm were adhered. The 1.2 mm thick CFRP tape manufactured by Sika CarboDur S (Sika Poland, Warsaw, Poland) was used to obtain the sections. The adhesives obtained during their modification according to the scheme described earlier were used to glue the tape sections to the concrete surface. Adhesion of adhesives on concrete surfaces prepared according to the following methods was analyzed:

- C—the surface of the concrete cleaned of dust, laitance and other fine dirt with a brush;
- G—concrete surface ground with a diamond disc;
- S—concrete surface wet-sanded with quartz sand with a diameter of 0.1–0.5 mm.

Before gluing, each sample was dried for 10 h at 105 °C to remove any moisture from the concrete surface. The presence of even a small amount of water on the surface of the tested concrete could effectively interfere with effective adhesion to its surface.

### 2.2. Methodology

The described research program involved the following tests:

- Measurement of viscosity of unmodified adhesive, measuring the temperature and viscosity of the resin when the sonicator was turned off, after 7 min of sonication;
- Measurement of the temperature and viscosity of the resin at the moment when the resin reached a comparative temperature of 22 °C, at which further processing of the adhesive was performed, at intervals of 5 min between successive measurements;
- Performing tests on the strength parameters of samples made from the described adhesives: surface hardness, tensile strength, elastic modulus, Poisson’s ratio;
- Manufacturing of class C30/37 concrete specimens constituting the substrate for re-gluing the CFRP tape fragments with the selected adhesive;
- Preparation of the surface of the samples according to the accepted methods of concrete surface treatment;
- Gluing the tape fragments to the concrete samples and testing the adhesion of the samples to the concrete substrate using a modified version of the pull-off test;
- SEM analysis of samples extracted from resins;
- Analysis of the obtained results.

#### 2.2.1. Physical Properties

During the tests, viscosity measurements were carried out on unmodified adhesive (for comparison) and adhesives subjected to modifications, according to the scheme described in Section 2.2. Because of the need to obtain accurate measurements, a rotating stationary viscosity meter type H (FungiLab, Barcelona, Spain), with R2 spindle and a PT-105 laboratory thermometer by Elmetron (Elmetron, Zabrze, Poland) were used. The spindle rotation speed was set at 100 rpm. The viscosity meter as well as the thermometer have a measurement accuracy of 0.1 mPa·s (viscosity meter) and 0.1 °C (thermometer), respectively. Three measurements of viscosity and temperature were performed for each modified batch.

#### 2.2.2. Surface Properties

**Figure 2:**
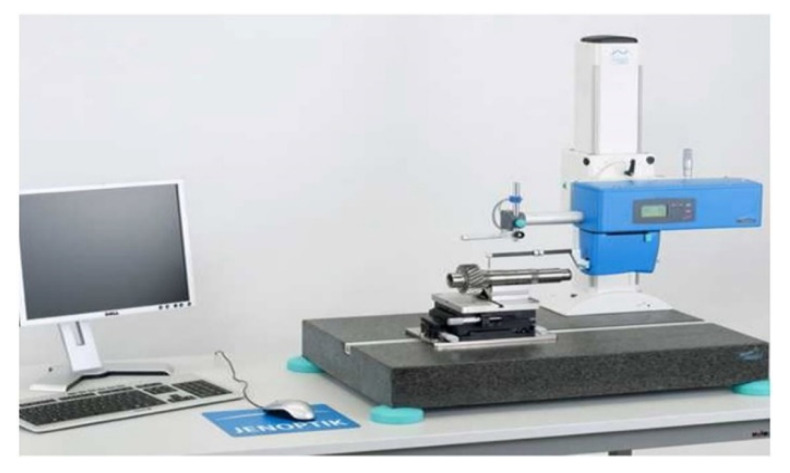
Test stand for profilometric testing.

Measurements were conducted for the cutouts obtained from the corresponding concrete surface. Each slice had dimensions of 5 × 5 cm. The operation of the profilometer consists of conducting 48 measurements through the movement of a needle in contact with the surface of the test specimen. The change in the position of the needle attached to the holder with the possibility of movement in the direction of the vertical axis is converted into electrical signals and, owing to a special module of the device, processed into an image of the tested surface. With the aid of the described device, it is possible to make a 2D and 3D profilogram. The measurement was carried out on 5 × 5 cm sections of the surface. As a result, a complete database of each surface and its roughness is obtained along with its image. The test stand is shown in Figure 2.

#### 2.2.3. Mechanical Properties

The concrete grade test was performed on a CONTROLS (Milan, Italy) test press with a load range of 0–3000 kN. The modulus of elasticity was determined in a WalterBai apparatus (Lohningen, Switzerland) with an attachment containing an electrofusion strain gauge and a programmed test. In order to check the correctness of the class designed for concrete, 5 cubic specimens with dimensions of 15 × 15 × 15 cm were prepared. The test of the modulus of elasticity of concrete was carried out on 3 cylindrical specimens with a diameter of 15 cm and a height of 30 cm, according to the standard guidelines [57].

**Figure 3:**
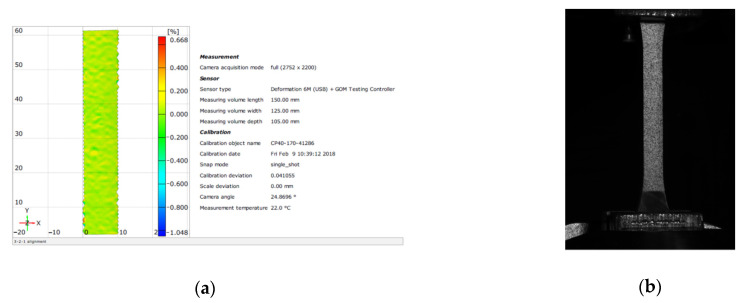
View of the specimen in the testing press and on the screen of ARAMIS software. (**a**) image of the specimen in the system; (**b**) specimen with applied pattern.

The tensile strength, modulus of elasticity and Poisson’s ratio of the adhesives were determined on 4 mm thick paddle-shaped specimens (Figure 3) [58,59]. For each series, six measurements were conducted using an MTS 810 strength tester (MTS Systems, Eden Prairie, MI, USA) with electronic recording with an attachment with a load range of up to 5 kN. A displacement control of 1 mm/min was used. The tool supporting the reading of axial tensile strength and the modulus of elasticity was a special ARAMIS system (Gom a Zeiss Company, Oberkochen, Germany). ARAMIS software enables one to conduct this type of analysis by using a non-contact, three-dimensional strain measurement and a 2D and 3D image analysis method. This measurement is made possible by locating changes in the position of points on the surface of the sample obtained by applying a special contrast-based pattern to the sample (Figure 3). A camera with very high resolution and sensitivity captures the first image, which serves as a comparison level for subsequent images in which individual points change their position during the test. During the described tests, the number of pictures taken is 500/min. The corresponding counting module then performs a comparative analysis of pixel position changes in successive photos. If the input data are given, i.e., the thickness of the specimen and the speed of change of the press piston displacement, it is possible to obtain the complete results of the strength parameters with their corresponding graphical presentation (Figure 3). All tests were carried out 14 days after the samples were molded and the resins hardened.

The hardness on the surface of the specimens of each series was determined using the Vickers method, taking 10 N as the base load. A Vickers multifunction hardness tester (ZwickRoell GmbH, Ulm, Germany) with a load range of 0–200 N was used. The result of a single measurement is formed by determining the dimensions of the diamond-shaped imprint left on the specimen by the blade of the pyramid-shaped measuring tip. Ten hardness readings were taken for each series. The device determines hardness as the ratio of the force exerted on the test surface of the sample to the surface of the imprint.

**Figure 4:**
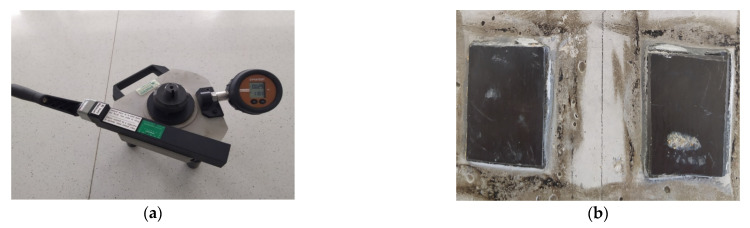
Pull-off testing device (**a**) and view of the CFRP tape pieces glued to the cleaned surface (**b**).

The adhesion of the adhesives to the various types of concrete substrate was determined by pulling off sections of CFRP strips adhered with the analyzed adhesives from the concrete (Figure 4b). For this purpose, a modified version of the pull-off test was used with an apparatus from Dynatest (Gainesville, FL, USA) having a load range of 0–25 kN (Figure 4a). Four measurements were conducted for each formulation. The average thickness of the adhesive layer between the CFRP tape and the concrete surface was 0.5–0.6 mm, which is due to the composition of the adhesive containing quartz flour. The age of the concrete to which the samples were glued ranged from 360 to 400 days. The assumptions made were intended to partially address the actual conditions of the substrate condition after a certain period of service. The value of the stress peeling the sample from the substrate was determined as the quotient of the peeling force and the area of the CFRP tape sample. All tests of the strength parameters of the adhesives were carried out 14 days after curing.

#### 2.2.4. Microstructural Properties

In the final stage of the conducted research, the structure of samples extracted from the surface of resins was analyzed using a scanning electron microscope (SEM)—Quanta 250 FEG (FEI, Hillsboro, OR, USA). The microscope is equipped with a LaB6 cathode electron gun, which, based on spectroscopy methods, enables one to obtain a very accurate image of the sample under study. The analysis was performed under high vacuum conditions. The samples were attached with carbon tape to aluminum holders before testing. The carbon electrically conductive layer, about 50 nm thick, was obtained via a sputtering process in a Quorum Q150T sputtering machine (Quorumtech, Laughton, UK). The test was carried out under secondary electron (SE) light, with an accelerating voltage in the range of 10^−15^ keV.

## 3. Results and Discussion

### 3.1. Physical Properties

#### Viscosity and Density of Unmodified and Modified Adhesive

**Figure 5:**
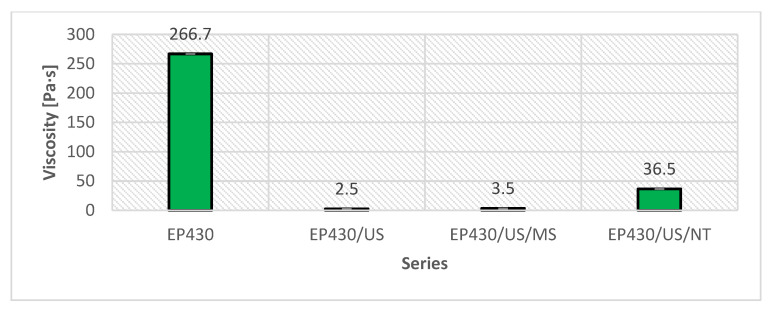
Results of viscosity measurements for the modified series when the sonicator was turned off.

**Figure 6:**
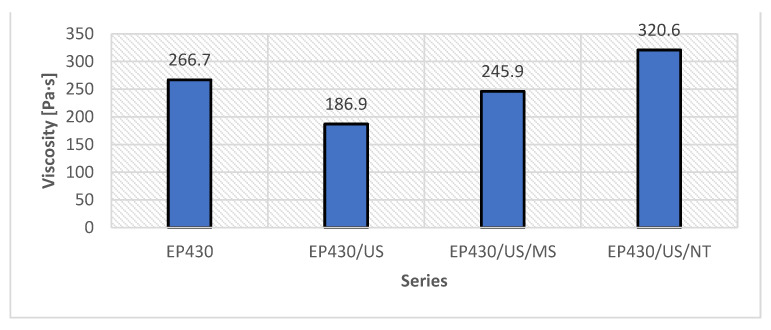
Viscosity measurement results for the unmodified and modified adhesive series.

The viscosity values of the adhesive when the sonicator was turned off, compared to the viscosity of the unmodified adhesive, are shown in Figure 5. In turn, Figure 6 shows the viscosity values after the adhesives cooled to 22 °C. No change in density was noted for any batch, which reached an average value of 2.05 g/cm^3^.

The phenomena affecting changes in the viscosity of individual ones result from the occurrence of ultrasonic cavitation [60,61,62,63]. This effect is based on the dynamic phenomena of liquid–gas phase transition caused by the occurrence of so-called cavitation media, usually in the form of bubbles containing oxygen, nitrogen, water vapor or light hydrocarbons. The local pressure in the bubble is higher than in the surrounding medium, in this case the adhesive mass. As a result of bubble implosion, there are changes in resin pressure and temperature. Similar phenomena were described in works [10,35,60,64] for other resins and cavitation parameters. The longer sonication time adopted in the described studies was due to the presence of quartz flour in the adhesive. Its particles are able to absorb the heat released during bubble bursting. The effects of cavitation in the form of an increase in temperature and a decrease in viscosity are prolonged in time; the heat is more evenly transferred between the different zones of interaction of the sonicator tip. As a result, as can be seen in the graph, it was possible to reduce the viscosity of the adhesive of the EP430/US formulation by 30.0%, the EP430/US/MS series by 7.8% and increase by 20% the last EP430/US/NT series. Each of these changes is due to the occurrence of certain common phenomena, which are connected with the effects of ultrasound and its induced sonication on the mass of epoxy resin and quartz flour [60]. During sonication, there is a reorganization of the original structure of the polymer associated with the relocation of the electron from monomers located in the electron clouds and the release of free radicals [1,2,3]. Similar to the appearance and implosion of cavitation bubbles [65], the occurrence of radicals is a short-lived process, as they seek to reconnect with other molecules. This task is facilitated by the presence of free electrons, which, owing to ultrasound, acquire an elevated energy state, leading to their faster exchange between monomers. As noted in [10,60,63], the electrons from the silica particles contained in the quartz flour are probably also involved in the exchange of electrons between molecules. This process allows the transfer not only of electrons on the atomic scale, but also of cavitation-induced heat on the macroscopic scale, leading to a decrease in viscosity and an increase in temperature. The result was low viscosity values, shown in Figure 5. Similar effects, with other adhesive and cavitation parameters, were also demonstrated in [10,35,60]. The release of radicals and electrons is also associated with the development of cluster structures, characteristic of polymers, which these materials adopt in equilibrium when not involved in chemical reactions [1,2,66]. Due to a certain orientation of the movement of molecules, which is caused by the propagation of ultrasonic waves in the medium, Brownian motions characteristic of such systems appear [1,14,66]. The viscosity changes shown in Figure 6 are the result of phenomena occurring during the cooling of adhesives and follow different patterns. In the case of the EP430/US series, during the return of the polymer to a temperature of 22 °C, a certain ordering of the structure takes place, binding the previously free bonds at the ends of the polymer chains. This is also the result of the formation of more bonds between molecules, which is related to the exchange of excited electrons. In the series modified with filler additives, it is possible to form bonds associated with van der Waals and London forces [1,2,14,60]. The microsilica can freely enter between the free spaces in the three-dimensional structure of the polymer. Since it is a different form of SiO_2_ than quartz flour, characterized by higher reactivity, electron exchange between the monomers and microsilica particles is possible. The spherical shape of its particles and the described interactions reduce viscosity, however, to a lesser extent than in the EP430/US series. Nevertheless, these processes occur quite differently for the EP430/US/NT series. While sonication only results in the activation of microsilica molecules without changing their phase state, carbon nanotubes are first broken down, followed by the straightening of their structure and the formation of a secondary network composed of carbon atoms. Between the atoms there are mainly single bonds; however, weaker double bonds are also present, which are first broken during sonication. Such a network is able to interpenetrate with the polymer network, linking to it through chemical bonds. This is also possible because carbon is the main element building the polymer chain of the epoxy resin contained in the adhesive. As a result, the structure of the polymer becomes much denser, which translates into an increase in its viscosity. With regard to the application of the adhesive under real conditions, both lowering and increasing the viscosity can be a beneficial phenomenon [16,19,35,60,67]. Lower viscosity provides the opportunity for the adhesive to penetrate rougher substrates more easily when, for example, bonding panels to substrates or applying protective layers. Higher viscosity, on the other hand, may allow greater initial adhesion of an adhesive applied while still in the liquid phase to a substrate with a less varied roughness profile.

### 3.2. Surface Properties

**Figure 7:**
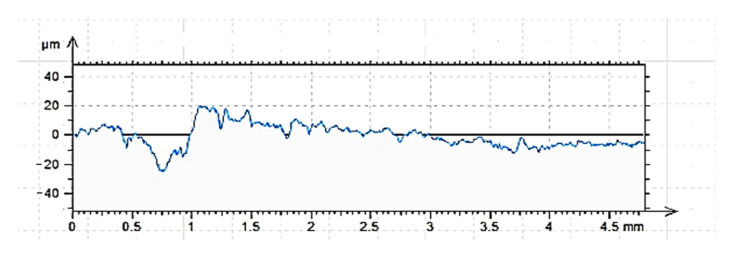
Cleaned surface roughness profile C, length = 4.80mm, Pt = 44.7 μm, Scale = 100.00 μm.

**Figure 8:**
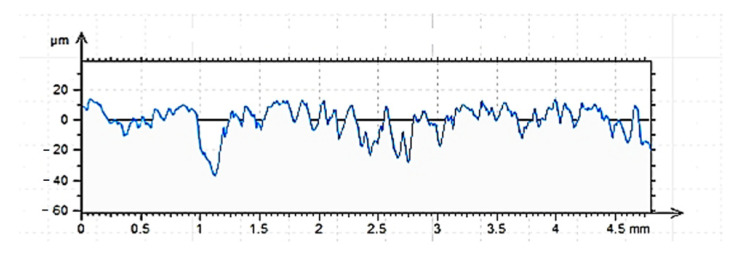
Sandblasted roughness profile G, length = 4.80mm, Pt = 51.0 μm, Scale = 100.00 μm.

**Figure 9:**
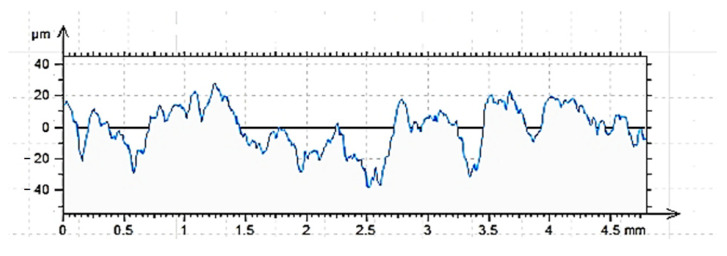
Sandblasted surface roughness profile S, length = 4.80mm, Pt = 66.6 μm, Scale = 100.00 μm.

The results of measurements (Figure 7, Figure 8 and Figure 9) of the vertical and amplitude parameters of the roughness profiles of the cleaned (C), ground (G) and sandblasted (S) surfaces, presented below, were reported in [60] for tests of a different type of adhesive for other applications. The results obtained are authoritative for various analyses. It was established that with the same type of concrete, its surface treatment and sampling for profilometric tests, there is a proper repeatability of the results. A summary of the values of individual vertical and amplitude parameters (introduction) is provided in Table 3.

The results of roughness tests performed by contact profilometry are extremely helpful in determining the adhesive performance of various building materials [15,18,45]. The effect of each of the surface preparation methods used is already visible in the images obtained in the form of roughness profiles. In this respect, the sandblasted surface showed the most varied roughness. The ground surface shows the most regular distribution of irregularities, while the surface prepared only by cleaning the top layer of concrete shows the least regularity in the occurrence of hills and depressions. These conclusions are also consistent with other analyses conducted for such prepared surfaces [45,60,68,69]. The shape of the roughness profile translates into specific results obtained for individual surfaces (Table 3). According to the nature of the roughness profile of the cleaned surface (C), the lowest absolute values of all vertical parameters were obtained, while the highest values were obtained for the sandblasted surface. The ground surface showed intermediate values, however, significantly higher than the cleaned surface. The differences between the cleaned and sandblasted surface are about twice as large in favor of surface G. The sandblasted surface does not differ in terms of values as much as the results for the ground surface. Characteristic for the surface prepared in this way are greater depths of individual depressions, while the ground surface is characterized by a more regular distribution of depressions (the most significant from the point of view of penetration of the adhesive into the irregularities of the substrate), which do not obtain such large single values. The greater roughness primarily affects the increase in mechanical adhesion between the adhesive and the substrate. However, this is not the only factor determining the effectiveness of the adhesive itself. From the group of amplitude parameters, R_a_ (the arithmetic mean deviation of the roughness profile) and R_q_ (the root mean square of the ordinates of the roughness profile) are most often used to evaluate roughness [15,43,44]. Ra allows obtaining a correlation between the results achieved for a relatively small sample and those occurring on larger surfaces. The use of this parameter enables elimination of individual kinks, pits and rises that occur on the surface of concrete. The cleaned surface is characterized primarily by these types of structures. As can be seen from Table 3, the highest Ra value was obtained by the sandblasted surface, followed by the ground surface (R_a_ lower by 18.6%). The cleaned surface, as in the case of vertical parameters, had the lowest R_a_, lower by 61.1% compared to the sandblasted surface and 51.1% compared to the ground surface, respectively. An auxiliary parameter for assessing roughness is also the value of R_q_, which, according to [43,44], takes a value about 25% higher than R_a_. For the described surfaces, this condition was met. It is also necessary to consider the values of the Rsk parameter, which reached negative values for all the described surfaces. This means that the tops of the hills are characterized by a relatively flat surface and the grooves are long and narrow. As the absolute value of the parameter increases, one can infer a more regular distribution of irregularities along the roughness profile, but also greater depths of the resulting depressions. Adhesive, due to the action of gravity and physicochemical phenomena, more easily fills depressions and irregularities than it covers hills. For the course of bonding, sharp peaks and hills are the most unfavorable, as they prevent effective coverage by the polymer layer. Confirmation of the R_sk_ results is also provided by the results of the R_ku_ parameter. With a value of the R_ku_ parameter < 3, the profile is characterized by a relatively small number of high, sharp elevations and deep depressions, as well as irregular defects [70]. The tops of the elevations are smoothed in this case. The penetration of the adhesive deep into the irregularities of the rough surface, in addition to the described amplitude parameters, is very much influenced by the width of the grooves, denoted as R_sm_. As can be seen, unlike the previous parameters, which clearly allowed the description and interpretation of a particular type of surface, the width of the grooves of the cleaned and sandblasted surface is very similar. The influence may be caused by the processes already described. Cleaning does not cause significant surface differentiation, but only allows the removal of layers that are weak and easily detached from the concrete surface. The surface layer itself remains relatively even, as is the case with a ground surface. Sandblasting clearly differentiates the roughness profile, which has the effect of more than doubling the value of the R_sm_ parameter. The greater width of the grooves may determine that the adhesive polymer more easily fills in the irregularities of the concrete surface. Regarding the value of R_dq_, it should be interpreted as follows. This parameter describes the slope of the roughness profile and determines the mean square of the slope of the ordinates in the base length [15,45,70]. Higher values of the parameter, obtained in the described studies for sandblasted and ground surfaces, result in higher interlaminar friction, less surface reflection, greater susceptibility to deformation during loading (e.g., under frictional conditions) and greater adhesion to the shaped substrate.

### 3.3. Mechanical Properties

#### 3.3.1. Tensile Strength, Elastic Modulus and Surface Hardness of Resins

Table 4 summarizes the results of all mechanical parameters of the tested EP430 adhesive formulations. For comparative purposes, the results for samples made from unmodified adhesive were also determined.

Analysis of the changes in the results of hardness tested on the surface of the samples showed the varying influence of the applied modifications on this parameter. The results give a preview of what properties the adhesive has in the contact layer with the substrate. Compared to the result obtained for the unmodified series, there was a decrease in hardness for the EP430/US and EP430/US/NT series by 6.5 and 20.0%, respectively. This fact may mean that the surface layer of the resins in these series is characterized by greater elasticity and deformability. The changes in hardness are a direct result of the phenomena described when discussing the viscosity results and related to the sonication of the resin in the liquid state. After curing, the effects in the form of varying HV10 values may be due to the different intensity and speed of the crosslinking process on the surface of the samples. Similar conclusions can be drawn from the analyses presented in [71,72]. Modification using only ultrasound does not significantly affect this process; however, the addition of nanotubes significantly reduces the hardness measured on the surface. This may also be related to some accumulation at the surface of the samples of agglomerates composed of nanotubes, which did not take part in the formation of connections with the polymer. However, the result obtained for the EP430/US/MS series was 14.3% higher than that of the EP430 series. This condition may be related to the conclusions described for other studies regarding the possibility of collecting microsilica particles near the surface of the sample, which leads to its strengthening [39,53,60]. In the case of this series, it is apparent that the crosslinking process proceeded more efficiently at the surface.

Differences in the values of tensile strength results may be due to similar dependencies. The organization of the polymer structure after sonication leads to a packed and ordered structure, which is characterized by molecules that react faster with the hardener [53,60,72]. This translates into a 65.8% higher tensile strength value. The other series showed a very slight increase in strength, by EP430/US/MS and EP430/US/NT series in the range of 1–3%. This shows that the proportion of fillers changes a number of processes related to the arrangement of polymer molecules. It also affects the crosslinking of the polymer. However, while the differences in surface hardness results were apparent, the introduced fillers did not cause a decrease in tensile strength values. However, the possibility of a different amount of fillers remains an issue for further clarification. As demonstrated by Majeed in [73], the amount of microsilica has only a limited beneficial effect on the strength parameters of the epoxy adhesive. Beyond a certain limit, the effect can be counterproductive. The results of tensile strength were reflected in the values of the elastic modulus. None of the analyzed series showed a decrease in this parameter (changes in the range of 1.0–5.6%). This means that the adhesive layer, after curing, should not lose its stiffness and durability even despite differences in surface hardness results. In this case, the sonication time, during which it was possible to completely mix the adhesive and fillers, reorganize them and activate the molecules for the possibility of forming chemical bonds, may have played a certain role [1,53,74]. Some variation in the results leads to the conclusion that the applied modification must be carefully considered in relation to the target application. Depending on the purpose for which the resin is used, i.e., bonding covering and finishing elements to wall or flat surfaces, making protective and utility coatings, it is important to carefully prepare the substrate and determine the assumed effect of using a given modification. The shape of the filler molecules is also important because it determines the way they are connected to the polymer chains. The longitudinal structure of the nanotubes, which break down under the influence of ultrasound, enables their specific adjustment to the shape of the polymer structure itself and the creation of interpenetrating networks. Thus, the expected direction of the tubes is identical to that of the polymers themselves. Microsilica particles, due to their spherical shape, may only be located in free spaces between polymer chains. The strength of the connection of filler molecules with the polymer results from the strength of intermolecular interactions and the formation of chemical bonds (permanent and temporary). The results shown in Table 5 give some idea of what load-bearing capacity and durability can be obtained by the adhesive subjected to the described modifications.

#### 3.3.2. Pull-Off Adhesion

The second stage of studying the strength parameters of the tested modified adhesive formulations was to determine its adhesion to the concrete surface. The results of these measurements are shown in Table 5, Table 6 and Table 7.

The analysis of the results assumed the determination of the contribution to the adhesive–substrate interface of mechanical and adsorptive adhesion, which in this case actually affects the total adhesion. In the case of f_b_ adhesion tested on the cleaned surface, its highest value was obtained for the unmodified series. The modified series showed a decrease in adhesion of 4.0% (EP430/US), 12.0% (EP430/US/MS) and as much as 52.0% (EP430/US/NT series), respectively. These results are primarily due to the low variation in the very surface on which the adhesive was tested. As a result of the poorly developed specific surface of such a surface, the effects for the series prepared in this way could not achieve the expected effect of increasing adhesion. Despite the fact that the adhesive binder of EP430 is epoxy resin, a material with relatively good adhesion to various materials [75,76], no modified series showed an increase in f_b_. The reason for this may also be the relatively low content of the binder itself, which is replaced by quartz flour in more than 60% of cases with regard to this adhesive. The contribution of mechanical adhesion is greater than that of adsorption, because the cleaned surface does not have the appropriate energy state caused by the concentration of charge-containing centers (electrons) that could participate in the formation of chemical bonds with the adhesive [60]. It should also be noted that in the case of pull-off adhesion to the cleaned surface, there is some correlation with the results of surface hardness.

A completely different relationship was shown by the measurements conducted on the ground surface. In this case, each modified series showed an increase in adhesion to the concrete surface—by 10.0% for the EP430/US and EP430/US/NT series and by 20% for the EP430/US/MS series. The condition of the ground surface, characterized by a higher specific surface area, as a result of the processing method adopted, is responsible for this [36,41,45,60]. Thus, adhesives, the ability of which to bond with the substrate is activated due to sonication and filler additives, are able to penetrate more easily into deeper irregularities of the substrate. The proportion of mechanical adhesion thus increases. However, there is also an increase in chemical bonds, mainly van der Waals interactions. Grinding exposes aggregate grains, in the described case granite and sand. According to the definition of the so-called Lewis acid-base theory, in such a system, the substrate is the electron donor (Lewis base) while the epoxy resin is the electron acceptor (Lewis acid) [50]. Grinding, in addition to increasing the roughness, also causes a significant increase in the energy state of the substrate [15,18,45]. The formation of bonds between the substrate and resin involves not only the molecules of the adhesive, but also the added fillers. In this case, attention should be paid to the EP430/US/MS series. In an earlier analysis, it was determined that the particles of this filler accumulate near the surface of the adhesive. In this way, they can bind to it more easily on the basis of the occurrence of chemical bonds, as well as better fill its irregularities by wedging and interlocking. Microsilica is a finer form of SiO_2_ in terms of fraction, so this effect is possible. In the case of nanotubes, the increased adhesion may be influenced by the higher viscosity of the adhesive when applied to the substrate (Table 3).

The f_b_ results obtained on the sandblasted surface showed an even greater effect of the applied modifications. As with the sanded surface, each modified series exhibited an increase in adhesion. Compared to the result obtained for the unmodified series, there was an increase in adhesion of 20.0% for the EP430/US series, 52.5% for the EP430/US/MS series and 17.5% for the EP430/US/NT series. This effect was, obviously, partly due to the greatest roughness of this type of substrate, which translated into an increased proportion of mechanical adhesion. Sandblasting, compared to grinding, does not expose the aggregate grains under the cement slurry layer; however, it also leads to activation of the substrate. This is associated with the removal of weak boundary layers [15,18,45,77]. Their detachment caused by the velocity of sand sanding the surface leads to a permanent substrate, which has distributed active centers containing electron groups. In this way, in addition to significant mechanical adhesion, it is possible to increase the share of adsorptive adhesion, as also demonstrated in [60]. The highest f_b_ value was achieved by samples of the EP430/US/MS series. In this case, the addition of microsilica is responsible for this condition, according to the same scheme as described for the ground substrate. When analyzing the results obtained in the pull-off test, it should be concluded that the proposed modifications to the EP430 adhesive are beneficial, provided that the substrate is properly machined. It is expected that other methods of surface preparation would also be beneficial for increasing the adhesion, the strength and, consequently, the durability of the adhesive layer.

### 3.4. SEM Analysis of Resin Structure

**Figure 10:**
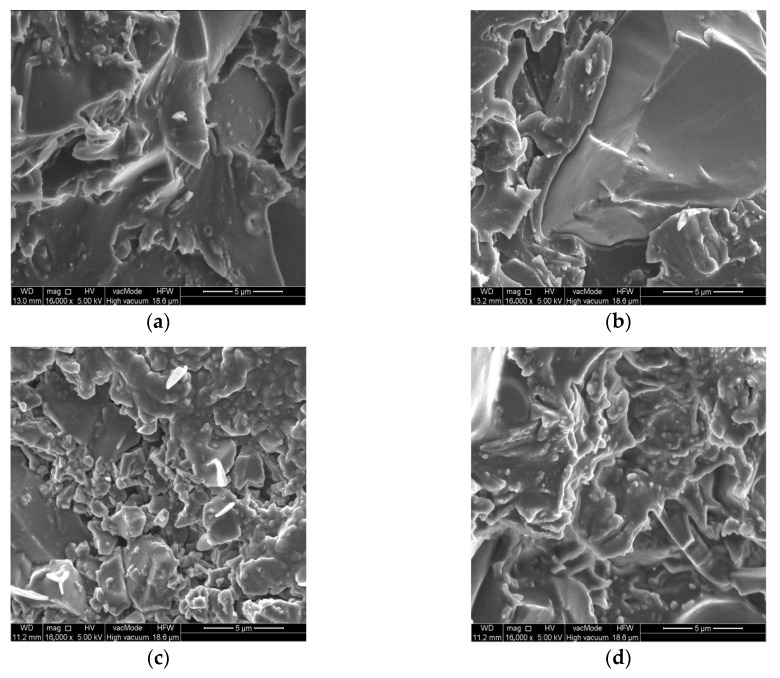
Breakthrough surface morphology of modified epoxy resins: (**a**) EP430; (**b**) EP430/US; (**c**) EP430/US/MS; (**d**) EP430/US/NT.

The last step of the study involved performing SEM analysis of the samples extracted from the surface of the resins. The images taken for each series are shown in Figure 10. The image shows some differences between the structures of the different series. Their presence is partly due to the original organization of the molecules. The structure for the EP430 series is chaotic and disordered. Greater orderliness is apparent for the EP430/US series. It is characterized by a large, flat region with a glassy structure. There are also the regions showing disrupted polymer chains, which can more easily disappear into the irregularities of the rough substrate. The image taken for the EP430/US/MS series sample shows lumpy clusters of material in many places, which were also found during the analyses presented in [11,53,60]. These characterize the microsilica clumped on the surface of the sample, confirming the earlier conclusion presented when discussing the HV10 hardness results. Their presence is due to the arrangement of quartz flour and microsilica in the resin structure. However, the behavior of quartz flour and microsilica during sonication or contact with the polymer may be different, because despite their identical chemical composition, the particles of these fillers show different form and chemical reactivity. The rough texture and the addition of quartz flour allow for better bonding to the concrete due to wedging of the flour grains into the irregularities of the substrate. The large variation of structures on the surface of the EP430/US/MS series allows better adaptation to the irregularities of the rough concrete substrate.

The chemical reactivity of the microsilica and the resin binder, meanwhile, allows for an increase in the proportion of adsorptive adhesion. The addition of microsilica as a reactive form of silicon dioxide was ultimately intended to create active zones that would more easily bind to the substrate as a result of altered electron distribution and surface topographical variation. The visible, irregular microstructures are characterized by a higher degree of dispersion than in the EP430/UD series. This suggests the possibility that the microsilica is partially bound to the polymer and distributed between the quartz flour particles. The topography and arrangement of the irregularities is different than for the previously described series.

The addition of carbon nanotubes slightly changed the picture of the EP430/US/NT series. It is likely that the network created by breaking up the nanotubes during sonication penetrates deeper into the adhesive after curing. However, the surface of the resin also shows great variation, which translates into increased adhesion to the modified concrete surface. The large variety of the structures formed on the resin surface allows it to be better matched to the diverse surface of the concrete. Moreover, the active zones of the resin containing clusters of electrons may be more easily connected with similar zones present on the substrate. In this case, their compaction is determined by the method of surface preparation. The properties of the binder itself, which is the epoxy resin, are of great importance. The nanotubes resulted in specifically shaped microstructures, different from those of the EP430/UD/MK series. Their shape is not as lumpy, nor is it characterized by rounded edges. Some of the hills have sharp edges, which may be due to the formation of temporary connections between quartz flour particles and nanotubes. The spherical SiO_2_ particles may provide the basis on which the nanotubes settle, mainly by shaping the van der Waals forces. The adhesive structure formed in this way allows easy adhesion to the substrate due to the presence of active zones in the described forms.

The presence of fillers in the resin structure can also bring another benefit. Their molecules can trap electrons in the adhesive, the release of which leads over time to the aging of the adhesive layer and its weakening. This happens as a result of the release of free radicals, which are reactive forms of monomer molecules. In a situation where there are microsilica or carbon nanotubes in the resin structure, they can “capture” the radicals immediately after their release and retain them in the polymer structure. In this way, aging is delayed, which also further affects the durability of the polymer on a given surface. It should be noted that the modified series showed greater adhesion to sandblasted and ground surfaces with increased roughness and the ability to increase mechanical adhesion. The values of adhesion measured on the surface that was only cleaned are also higher, which indicates an increased share of adsorption adhesion resulting mainly from the movement of electrons between the adhesive and the substrate.

### 3.5. Correlation Analysis

In Table 8, the correlation matrix between the examined values is presented. Two correlation coefficients with conventionally accepted markings, Pearson (r) and Spearman (ρ), were included in the correlation analysis. Their application enables the determination of the strength of the correlation and its type according to the key linear correlation (Pearson) and non-linear correlation (Spearman). The comparison of the values of both coefficients allows one to determine whether the relationship is more linear or non-linear.

The analysis of global relationships shows the existence of both linear and non-linear correlations. The relations between the modulus of elasticity and the tensile strength are the most linearly correlated (|r| = 0.94). This is consistent with the general nature of these quantities, which are closely related to each other as parameters describing the behavior of the tensile sample. There is also a strong correlation between the viscosity and the tensile strength and the modulus of elasticity (corresponding |r| = 0.83 and 0.82). In this case, however, it is difficult to speak of an effective explanation of this state. The viscosity of the adhesive was measured in its liquid state, while the mechanical parameters relate to the properties of the adhesive after curing. Moreover, a fairly strong correlation was found between the surface hardness and the pull-off adhesion tested on the cleaned concrete surface (|r| = 0.78).

Among the nonlinear correlations, a strong relationship was found between the tensile strength, adhesion on the cleaned and ground surface, and hardness, (|r| = 0.8). Additionally, between the Young’s modulus and the fb of the ground surface there is virtually complete correlation (|*ρ*| = 0.94), but this indicates the occurrence of a non-linear relationship rather than a linear one. The remaining correlations do not show any significant significance, being characterized by lower values of correlation coefficients.

## 4. Conclusions

As a result of the literature analysis, the obtained findings of the conducted research and the interpretation performed, the following final conclusions were drawn:

- The changes in viscosity of the analyzed series were directly due to the type of modifications used. Depending on the condition and type of the substrate and its roughness, it is possible to select the modified adhesive in such a way as to facilitate its penetration into the irregularities of the substrate (on a rough surface).
- Substrate roughness significantly affects the variation of adhesive adhesion results. The significant development of the specific surface of the concrete, which translates into significant roughness, is of great importance. This results in an increase in the proportion of mechanical adhesion. The surface treatment itself also leads to activation of the surface in terms of the distribution of centers containing electron clusters, which can form chemical bonds with the adhesive layer.
- The proportion of adsorption adhesion depends on the type of modification and the substrate. It was significantly higher on the ground and sandblasted surface. Sonication used as a modification and a way to distribute filler molecules in the adhesive leads to reorganization of electrons, which more easily participate in charge exchange with the substrate.
- The EP430/US, EP430/US/MS and EP430/US/NT series were characterized by increased adhesion to concrete substrates modified by mechanical treatment.
- The correlation analysis carried out showed the existence of strong correlations between the mechanical properties of the series studied.
- The described modifications can be successfully used under conditions of practical application in the case of facade cladding, terraces and balconies.

## Figures and Tables

**Table 1:** Properties of the glue used in tests.

Resin	EP430
Form	gray mass with the addition of quartz flour
Flashpoint (°C)	170
Gelation time (min)	120
Epoxy number (mol/100 g)	<700
Density (in 22 °C) (g/cm^3^)	2.05
Viscosity (in 22 °C) (Pa·s)	200
Solubility	ketones, esters, alhohols
Chemical resistance to	tap water, sodium hydroxide, hydrochloric acid, concentrated hydrochloric acid, sulfiric acid, nitric acid, acetic acid, xylene, ethanol

**Table 2:** Recipes and series used in research.

Series	Resin Type	Type of Additive/Modification	Amount of Filler (%)	Amount of Hardener (%)
EP430	epoxy	—	—	3
ER430/US		sonication	—	3
ER430/US/MS		sonication + microsilica	0.5	3
EP430/US/NT		sonication + carbon nanotubes	0.1	3

**Table 3:** Vertical and amplitude parameters of roughness profiles for concrete surface types.

Surface	R_p_	R_v_	R_z_	R_c_	R_t_	R_a_	R_q_	R_sm_	R_sk_	R_ku_
C	7.04	7.9	14.9	7.2	26.5	2.77	3.46	0.144	−0.107	3
G	12.6	17.1	29.7	16	45	5.66	6.96	0.149	−0.421	2.7
S	15.5	21.1	36.5	22.6	47.4	6.95	8.74	0.3	−0.467	2.89

**Table 4:** Test results of strength parameters of adhesives; ν—coefficient of variation.

Series	EP430	ν* (%)	EP430/US	ν (%)	EP430/US/MS	ν (%)	EP430/US/NT	ν (%)
**Hardness HV10**	23.0	2.0	21.5	3.0	26.3	2.1	16.0	1.8
**Tensile strength f_t.ax_ (MPa)**	19.6	3.4	32.5	2.8	20.1	2.5	19.8	1.5
**Elasticity modulus E_t_ (GPa)**	8.8	1.6	9.3	2.3	9.0	3.4	8.9	1.2

ν*—coefficient of variation.

**Table 5:** Results of pull of test for cleaned surface (C).

Series	Force (kN)	Pull-Off Stress (MPa)	Coefficient of Variation (%)
EP430	2.5	3.3	1.0
EP430/US	2.4	3.2	1.0
EP430/US/MS	2.2	2.9	4.1
EP430/US/NT	1.2	1.6	3.2

**Table 6:** Results of pull of test for ground surface (G).

Series	Force (kN)	Pull-Off Stress (MPa)	Coefficient of Variation (%)
EP430	2.8	3.7	1.2
EP430/US	3.1	4.1	3.0
EP430/US/MS	3.4	4.5	1.4
EP430/US/NT	3.1	4.1	4.8

**Table 7:** Results of pull of test for sandblasted surface (S).

Series	Force (kN)	Pull-Off Stress (MPa)	Coefficient of Variation (%)
EP430	3.0	4.0	2.1
EP430/US	3.6	4.8	3.1
EP430/US/MS	4.5	6.1	1.3
EP430/US/NT	3.5	4.7	3.6

**Table 8:** A matrix of Pearson’s and Spearman’s correlation coefficients. Designation of correlation strength: |r|<0.2—weak correlation; |r|∈⟨0.2÷0.4)—low correlation; |r|∈⟨0.4÷0.6)—moderate correlation; |r|∈⟨0.6÷0.8)—high correlation; |r|∈⟨0.8÷0.9)—very high correlation; |r|∈⟨0.9−1.0⟩—correlation virtually complete.

	Pearson’s Correlations (r)							
	Viscosity	HV10	f_t.ax_	E_t_	f_b C_	f_b G_	f_b S_	
Spearman’s correlations (ρ)	Viscosity	---	−0.54	−0.83	−0.82	−0.78	−0.15	0.49
	HV10	0.40	---	−0.02	0.09	0.78	0.31	0.49
	f_t.ax_	−0.40	0.80	---	0.94	0.38	0.03	−0.04
	E_t_	0.40	0.40	0.40	---	0.62	−0.51	−0.33
	f_b C_	0.01	0.80	−0.02	0.09	---	0.31	0.60
	f_b G_	−0.60	0.80	0.94	0.94	0.38	---	−0.04
	f_b S_	0.40	0.40	0.40	0.38	0.28	0.38	---

## Data Availability

Not applicable.
